# Molecular characterization and new genotypes of *Enterocytozoon bieneusi* in minks (*Neovison vison*) in China

**DOI:** 10.1051/parasite/2018038

**Published:** 2018-07-20

**Authors:** Wei Cong, Si-Yuan Qin, Qing-Feng Meng

**Affiliations:** 1 Marine College, Shandong University at Weihai Weihai Shandong Province 264209 PR China; 2 General Station for Surveillance of Wildlife Diseases & Wildlife Borne Diseases, State Forestry Administration (SFA) Shenyang 110034 PR China; 3 Jilin Inspection and Quarantine Technology Center Changchun Jilin Province 130062 PR China; 4 College of Animal Science and Technology, Jilin Agricultural University Changchun Jilin Province 130118 PR China

**Keywords:** *Enterocytozoon bieneusi*, Epidemiology, Genotyping, Minks, China

## Abstract

Microsporidiosis is an emerging and opportunistic disease, and *Enterocytozoon bieneusi* is the main cause of this disease in humans. Little information is available on prevalence and genotyping of *E. bieneusi* in minks. We collected 559 feces samples of minks from Heilongjiang and Jilin provinces in 2017, and studied *E. bieneusi* prevalence by nested PCR. A total of 23 out of 559 minks (4.1%) were detected as *E. bieneusi*-positive, and were raised in five of the seven investigated farms. Age was the only risk factor associated with *E. bieneusi* prevalence in investigated minks through logistic regression analysis. Sequence analysis of the ITS gene revealed that five *E. bieneusi* ITS genotypes, including Peru11, EbpC, and three novel genotypes (HLJM-1, HLJM-2 and JLM-1) were present, suggesting minks may be a potential source of human microsporidiosis.

## Abstract (fr)

La microsporidiose est une maladie émergente et opportuniste, et *Enterocytozoon bieneusi* est la principale cause de la microsporidiose humaine. Peu d’informations sur la prévalence et le génotypage d’*E. bieneusi* chez les visons sont disponibles. 559 échantillons d’excréments de visons provenant des provinces de Heilongjiang et de Jilin ont été collectés en 2017 et la prévalence d’*E. bieneusi* a été étudiée par PCR nichée. Au total, 23 (4,1%) des 559 visons ont été détectés positifs pour *E. bieneusi*, et provenaient de cinq des sept fermes étudiées. L’âge était le seul facteur de risque, étudiés par analyse de régression logistique, associé à la prévalence d’*E. bieneusi* chez les visons. L’analyse de la séquence du gène ITS a révélé que cinq génotypes ITS d’*E. bieneusi*, y compris Peru11, EbpC, et trois nouveaux génotypes (HLJM-1, HLJM-2 et JLM-1), étaient présents, ce qui suggère que les visons pourraient être une source potentielle de microsporidiose humaine.

## Introduction

Minks are a species of high economic importance: the animals are widely raised for their fur in Northern China, including in Heilongjiang and Jilin provinces. Minks can serve as reservoirs for many pathogens including influenza viruses [7], Aleutian mink disease virus [17], thrombocytopenia syndrome virus [21], *Pentatrichomonas hominis* [14], and *Toxoplasma gondii* [30]. Because minks are in close contact with their feeders, they can transmit many pathogens to humans, including *Toxoplasma gondii* [30]. Despite this, data regarding the prevalence and genotypes of *Enterocytozoon bieneusi* in minks are scarce.

The Microsporidia contains over 1300 named species, and has a worldwide distribution. *Enterocytozoon bieneusi* is the most frequent causative agent of human microsporidiosis [1, 11] and is responsible for more than 90% of human infections [3]. *E. bieneusi* can infect a variety of invertebrates and vertebrates [27, 28, 33], and can be transmitted through the anthroponotic, zoonotic, water-borne, and/or food-borne routes [4, 13, 19]. The symptoms of microsporidiosis caused by *E. bieneusi* are diarrhea and abdominal pain in immunodeficient individuals, while the infection appears asymptomatic in immunocompetent individuals who can shed spores into the environment and become a potential source of infection for other individuals [29].

More than 240 *E. bieneusi* genotypes have been defined based on the internal transcribed spacer (ITS) region of the rRNA gene [1]. All the genotypes can be grouped into 9 groups (named groups 1–9). The majority of human infections are caused by the zoonotic group 1 [6, 15]. However, some genotypes (such as I, J and BEB4) from the other zoonotic groups have also been found in humans [8].

In order to determine whether minks can be infected by *E. bieneusi*, and to assess the zoonotic risk of *E. bieneusi* between minks and humans, a total of 559 mink feces samples were collected from seven farms in Heilongjiang and Jilin provinces. The samples were tested to detect the prevalence of *E. bieneusi* and associated genotypes in minks by nested PCR amplification of the ITS region of *E. bieneusi*.

## Materials and methods

### Ethics statement

All animals were handled in strict accordance with good animal practices according to the Animal Ethics Procedures and Guidelines of the People’s Republic of China, and the study was approved by the Ethics Committee of Jilin Agricultural University.

### Specimen collection

In all, 559 farmed mink fecal samples were randomly collected from Heilongjiang (43°26′~53°33′ N, 121°11′~135°05′ E) and Jilin (41°~46° N, 122°~131° E) provinces, northeastern China in 2017. More than 200 minks were bred at each farm, and the sampling percentage ranged from 5% to 10% on the different farms. Fresh dejections were immediately collected using a polyethylene glove, and were then stored in ice boxes and transported to the laboratory. The Farm ID, gender and age of minks were obtained from the owners.

### DNA extraction and PCR amplification

The commercial E.Z.N.A.^®^ Stool DNA Kit (Omega Biotek Inc., Norcross, GA, USA) was used to extract genomic DNA, following the manufacturer’s instructions, and extracted DNA was stored at −20 °C. PCR targeting the ITS region was used to explore the prevalence and genotypes of *E. bieneusi*. All the PCR operations have been described in a previous study [31]. In each trial, positive and negative controls were present. The amplification products were observed using UV light after electrophoresis in a 1.5% agarose gel containing GoldView^TM^ (Solarbio, Beijing, China).

### Sequence and phylogenetic analyses

Sangon Biotech Company (Shanghai, China) was contracted to sequence the PCR products. Sequence accuracy was evaluated by bidirectional sequencing. Replicates were made when new sequences were found (single nucleotide substitutions, insertions or deletions). ClustalX 1.83 was used to align the sequences. The neighbor-joining (NJ) method (Kimura 2-parameter model, 1000 replicates) was used to reconstruct the phylogenetic trees with Mega 5.0 software. Representative nucleotide sequences were deposited in GenBank under accession numbers MH052578–MH052582.

### Statistical analysis

Data analysis of the prevalence of *E*. *bieneusi* infection in minks by age, gender, and different farms groups was performed by χ^2^ testing using SAS version 9.1 (SAS Institute, Cary, NC, USA) [16, 32]. When *p* < 0.05, the results were considered statistically significant. Odds ratios (ORs) and their 95% confidence intervals (95% CIs) were estimated to explore the strength of the association between *E*. *bieneusi*-positivity and the conditions investigated.

## Results and discussion

**Fig. 1.**
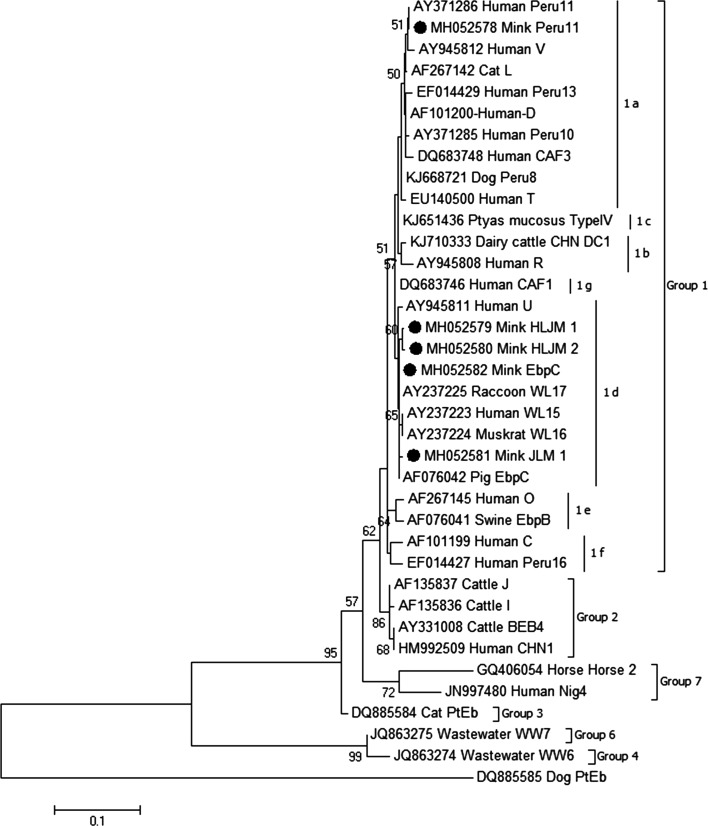
Phylogenetic analyses of *Enterocytozoon bieneusi* using the neighbor-joining (NJ) method (Kimura 2-parameter model). Bootstrap values below 50% from 1000 replicates are not shown. *E. bieneusi* isolates identified in the present study are pointed out by solid circles.

In the present study, 23 out of 559 minks (4.1%) were tested as *E. bieneusi*-positive. Female minks had a lower *E. bieneusi* infection rate than males (Table 1). The prevalence of *E. bieneusi* was 3.9% in minks from Heilongjiang Province, and 4.3% in minks from Jilin Province (Table 1). Minks aged more than three months had a significantly higher infection rate than those aged less than three months (Table 1). The *E. bieneusi* prevalence in the different investigated farms ranged from 0% to 7.5% (Table 2). Sequence analysis of the ITS region revealed that five *E. bieneusi* ITS genotypes (two known genotypes Peru11 and EbpC; three novel genotypes HLJM-1, HLJM-2 and JLM-1) were present in investigated minks (Fig. 1).

**Table 1.** Prevalence of *Enterocytozoon bieneusi* in minks in Jilin and Heilongjiang provinces, China.

Factor	Category	No. tested	No. positive	Prevalence (%) (95% CI)	*p*-value	OR (95% CI)
Region	Heilongjiang Province	257	10	3.9% (1.5–6.3)	0.81	Reference
	Jilin Province	302	13	4.3% (2.0–6.6)		1.1 (0.5–2.6)
Gender	Female	279	9	3.2% (1.2–5.3)	0.29	0.6 (0.3–1.5)
	Male	280	14	5.0% (2.4–7.6)		Reference
Age	≤3 months	244	5	2.0% (0.3–3.8)	0.03	Reference
	>3 months	315	18	5.7% (3.2–8.3)		2.9 (1.1–7.9)
Total		559	23	4.1% (2.5–15.8)

**Table 2.** Distribution of *Enterocytozoon bieneusi* genotypes on different farms.

Farm ID	Sample size	Prevalence (%)	Genotype (No.)
1	124	3.2	HLJM-1 (1); Peru11 (3)
2	133	4.5	HLJM-1 (1); HLJM-2 (1); Peru11 (4)
3	93	7.5	Peru11 (4); EbpC (3)
4	32	0	–
5	75	6.7	Peru11 (2); EbpC (3)
6	78	0	–
7	24	4.2	JLM-1 (1)
Total	559	4.1	Peru11 (13); EbpC (6); HLJM-1 (2); HLJM-2 (1); JLM-1 (1)

The overall infection rate of *E. bieneusi* in minks was 4.11%, which was higher than that in domestic rabbits (0.9%, 4/426) [32] in Jilin, pet chinchillas (3.6%, 5/140) [18] in Beijing, Zhengzhou, Anyang and Guiyang, and similar to the 4.1% infection rates in raccoon dogs (2/49) [29] in Liaoning, Heilongjiang and Jilin, and 4.6% prevalence in captive snakes (11/240) [10] in Guangxi. However, this rate was significantly lower than that in captive golden snub-nosed monkeys (46.2%, 74/160) [26] in Beijing, Shanghai, Anhui and Shanxi, and captive Asiatic black bears (27.4%, 29/106) [24] in Sichuan and Guizhou. The difference in *E. bieneusi* prevalence may be related to feeding conditions, sampling time, sample sizes, and animal husbandry practices, as well as variable susceptibility of different animals. *Enterocytozoon bieneusi* is a commonly enteric pathogen, and also exists extensively in the environment. *Enterocytozoon bieneusi* accumulation could occur throughout a minks lifetime. Therefore, minks aged more than 3 months (OR = 2.9, 95% CI 1.1–7.9, *df* = 1, *p* = 0.03) were at a 2.9 times higher risk of *E. bieneusi* infection compared to minks less than three months of age.

More than 50 *E. bieneusi* ITS genotypes have been found in captive animals in China [2, 5, 9, 10, 12, 18, 22–26, 29, 31, 32]. However, only five *E. bieneusi* ITS genes (two known genotypes, Peru11 and EbpC; and three novel genotypes, HLJM-1, HLJM-2 and JLM-1) were found in the present research (Table 2). Genotype Peru11 (distributed on four farms) was the most frequently found genotype of all four genotypes, and was responsible for 56.5% of all infections; genotype EbpC (*n* = 6, 26.1%) and HLJM-1 (*n* = 2, 8.7%) were found on two farms; HLJM-2 (*n* = 1, 4.3%) and JLM-1 (*n* = 1, 4.3%) were only identified on farm 2 and farm 7, respectively (Table 2). These findings suggest that the five genotypes were prevalent in investigated minks in Heilongjiang and Jilin, China. The Peru11 genotype was also found in captive non-human primates, laboratory macaques, and EbpC was prevalent in nonhuman primates and blue foxes in China [5, 9, 12, 18, 22–26, 29, 31, 32], which suggests that *E. bieneusi* may be transmitted among these captive animals.

The ITS sequence analysis demonstrated that the sequence of Accession Nos. MH052578 and MH052582 was identical to that of genotypes Peru11 (Accession No. KT922238) and EbpC (Accession No. KX905207), respectively. Moreover, all the five identified *E. bieneusi* genotypes were grouped into group 1 (Fig. 1). Peru11 was sub-classified into group 1a (Fig. 1); EbpC, HLJM-1, HLJM-2 and JLM-1 were sub-classified into group 1d (Fig. 1). More importantly, Peru11 and EbpC were also found in HIV-positive patients in Henan [20]. These findings suggest that minks may play an important role in human infections. Although no evidence of human microsporidiosis outbreaks originating from minks or other animals has been found, we should pay close attention to nosocomial transmission among humans, minks and other animals.

## Competing interests

The authors declare that they have no competing interests.
